# Supplementary material for: Construct and criterion validity of the HEXACO Medium School Inventory Extended (MSI-E)
Source: PLoS One. 2023 Oct 13;18(10):e0292813. doi: 10.1371/journal.pone.0292813 (PMC10575544; doi:10.1371/journal.pone.0292813)
Supplement: S1 File — (DOCX) [file pone.0292813.s001.docx]

**S1 File. Supplementary Materials**

These Supplementary Materials must be regarded as integral part of the paper:

Mottola, F., Abbamonte, L., Ariemma, L., Gnisci, A., Marcone, R., Millefiorini, A., Perugini, M., Senese, V.P., Sergi, Construct and criterion validity of the HEXACO Medium School Inventory Extended (MSI-E).

**Table A.**

Correlations among the six factors extracted from the facets (CFA) of the Obs HEXACO-MSI-E.

| Factor | H | E | X | A | C |
| --- | --- | --- | --- | --- | --- |
| H |  |  |  |  |  |
| E | .436 |  |  |  |  |
| X | .075 | -.433 |  |  |  |
| A | .606 | .227 | .374 |  |  |
| C | .409 | .102 | .353 | .302 |  |
| O | .145 | .033 | .356 | .193 | .616 |

*Note.* H = Honesty/Humility; E = Emotionality; X = Extraversion; A = Agreeableness; C = Conscientiousness; O = Openness to Experience.

**Table B.** Correlation matrix between the six HEXACO-MSI-E traits and (a) the self-report (*N* = 388) and (b) the reported by an observer youth emotional and behavioral problems (*N* = 373)

|  | **(a) Self-Report Criteria** | | | | | | | | **(b) Observer Criteria** | | | | | |
| --- | --- | --- | --- | --- | --- | --- | --- | --- | --- | --- | --- | --- | --- | --- |
|  | **HEXACO-MSI-E** | | | | | | | | **HEXACO-MSI-E** | | | | | |
| **Youth Emotional and Behavioral Problems** | **H** | **E** | **X** | **A** | **C** | **O** |  | **H** | | **E** | **X** | **A** | **C** | **O** |
| ***Broad-band scales*** |  |  |  |  |  |  |  |  | |  |  |  |  |  |
| Internalizing | -.16^*^ | .33^*^ | -.76^*^ | -.33^*^ | -.27^*^ | -01 |  | -.03 | | .06 | -.47^*^ | -.14^*^ | -.14^*^ | -.04 |
| Externalizing | -.59^*^ | -.15^*^ | -.38^*^ | -.62^*^ | -.53^*^ | -.27^*^ |  | -.32^*^ | | -.10 | -.19^*^ | -.32^*^ | -.30^*^ | -.14^*^ |
| ***Syndrome scales*** |  |  |  |  |  |  |  |  | |  |  |  |  |  |
| Anxious/Depressed | -.12^*^ | .42^*^ | -.69^*^ | -.26^*^ | -.23^*^ | .03 |  | -.04 | | .13^*^ | -.39^*^ | -.13^*^ | -.10 | -.02 |
| Withdrawn/Depressed | -.13^*^ | .17^*^ | -.77^*^ | -.35^*^ | -.26^*^ | -.04 |  | .01 | | -.03 | -.45^*^ | -.10 | -.13^*^ | -.07 |
| Somatic Complaints | -.25^*^ | .17^*^ | -.47^*^ | -.28^*^ | -.24^*^ | -.06 |  | -.11 | | .02 | -.27^*^ | -.13^*^ | -.12^*^ | -.01 |
| Delinquent Behavior | -.52^*^ | -.22^*^ | -.27^*^ | -.41^*^ | -.45^*^ | -.24^*^ |  | -.21^*^ | | -.05 | -.08 | -.09 | -.15^*^ | -.13^*^ |
| Aggressive Behavior | -.55^*^ | -.10 | -39^*^ | -.66^*^ | -.51^*^ | -.25^*^ |  | -.32^*^ | | -.11 | -.21^*^ | -.36^*^ | -.32^*^ | -.12^*^ |

*Note.* ^*^ FDR corrected p < .05 within each table.

**Table C.** Correlation matrix between the six HEXACO-MSI-E traits and (a) the self-report (*N* = 385 if not otherwise indicated) and (b) the reported by an observer youth risk behaviors (*N* = 373 if not otherwise indicated)

|  | **(a) Self-Report Criteria** | | | | | |  | **(b) Observer Criteria** | | | | | |
| --- | --- | --- | --- | --- | --- | --- | --- | --- | --- | --- | --- | --- | --- |
|  | **HEXACO-MSI-E** | | | | | |  | **HEXACO-MSI-E** | | | | | |
| **Youth Risk Behaviors** | **H** | **E** | **X** | **A** | **C** | **O** |  | **H** | **E** | **X** | **A** | **C** | **O** |
| When you ride a bike, skateboard or overboard (etc.), how often do you wear a helmet?^a, c^ | .20^*^ | .15^*^ | .21^*^ | .31^*^ | .47^*^ | .32^*^ |  | .06 | .01 | .24^*^ | .18^*^ | .17^*^ | .14 |
| How often do you wear a seat belt when riding in a car? | .15^*^ | .09 | .10 | .15^*^ | .29^*^ | .21^*^ |  | .07 | .04 | .12 | .13 | .14^*^ | .12 |
| Have you ever ridden in a car driven by someone who had been drinking alcohol? | -.13^*^ | -.01 | -.07 | -.15^*^ | -.10 | -.10 |  | -.05 | -.10 | -.04 | -.03 | -.05 | -.03 |
| Have you ever carried a weapon, such as a gun, knife, or club? | -.26^*^ | -.11 | -.07 | -.12^*^ | -.04 | .02 |  | .01 | -.03 | .01 | .02 | .05 | -.03 |
| Apart from a few sips, have you ever had a whole alcoholic drink? | -.29^*^ | -.10 | -.02 | -.16^*^ | -.18^*^ | -.11 |  | -.10 | -.02 | .05 | -.02 | -.08 | -.06 |
| Have you ever used marijuana for a relatively continuous period? | -.13^*^ | -.03 | -.03 | -.01 | -.05 | -.02 |  |  |  |  |  |  |  |
| How old were you when you first tried smoking a cigarette, even just one or two puffs? | .16^*^ | .08 | .08 | .08 | .08 | .15^*^ |  |  |  |  |  |  |  |
| In the last 30 days, on the days you smoked, how many cigarettes (regular or electronic) per day have you smoked? | .00 | -.08 | .00 | -.04 | -.06 | -.06 |  | - | - | - | - | - | - |
| Have you ever engaged in self-harming behaviors (cutting yourself, scratching yourself, etc.) on a voluntary basis? | -.14^*^ | -.06 | -.24^*^ | -.14^*^ | -.10 | -.01 |  | .02 | -.07 | -.15^*^ | -.12 | -.19^*^ | -.07 |
| Have you ever been involved in a physical fight (e.g., you got into a fight, brawls, etc.)? | -.21^*^ | -.26^*^ | -.03 | -.15^*^ | -.25^*^ | -.19^*^ |  | -.07 | -.10 | -.01 | -.06 | -.09 | -.11 |
| Have you ever been bullied at school? | -.05 | .06 | -.23^*^ | -.11 | -.10 | .04 |  | .02 | .04 | -.22^*^ | -.09 | -.16^*^ | -.03 |
| Have you ever been electronically bullied (through texting, Instagram, Facebook, or other social media)? | -.08 | .04 | -.21^*^ | -.08 | -.18^*^ | .01 |  | .00 | -.07 | -.20^*^ | -.09 | -.13^*^ | .07 |
| During the past 7 days, how many days have you eaten breakfast? | .07 | .09 | .19^*^ | .08 | .16^*^ | .12^*^ |  | .14^*^ | .03 | .10 | .07 | .12 | .06 |
| When you have class, how many hours do you sleep per night? | .12^*^ | .19^*^ | .17^*^ | .20^*^ | .23^*^ | .15^*^ |  | .18^*^ | .01 | .12 | .16^*^ | .28^*^ | .13 |
| On a typical school day (even if in distance learning), how many hours a day do you watch TV?^b, d^ | -.20^*^ | -.03 | .09 | -.09 | -.12 | .04 |  | .03 | .08 | .02 | .01 | -.06 | -.03 |
| On a typical school day (even if in distance learning), on average, how many hours a day do you play video games or use computer or mobile phone for things not related to school? | -.16^*^ | -.02 | -.11 | -.18^*^ | -.21^*^ | -.06 |  | -.15^*^ | -.01 | -.12 | -.13 | -.26^*^ | -.22^*^ |

For self-report criteria: ^a^n = 306; ^b^n = 299. For Observer crireria: ^c^n = 241; ^d^n = 321.

^*^ FDR corrected p<.05 within each table.

Note. Criteria are presented only in self-report form. In the observer form, they were presented in third person with the subject ‘your son/daughter’.

**Table D.** Correlation matrix between the six HEXACO-MSI-E traits and adolescents’ (a) self-report (*N* = 382) and reported by an observer values, beliefs, and behaviors (*N* = 373)

|  | **(a) Self-Report Criteria** | | | | | | | **(b) Observer Criteria** | | | | | |
| --- | --- | --- | --- | --- | --- | --- | --- | --- | --- | --- | --- | --- | --- |
|  | **HEXACO-MSI-E** | | | | | | | **HEXACO-MSI-E** | | | | | |
| **Adolescents’ values, beliefs, and behaviors** | **H** | **E** | **X** | **A** | **C** | **O** |  | **H** | **E** | **X** | **A** | **C** | **O** |
| I believe in God. | .10 | .16^*^ | .14^*^ | .17^*^ | .15^*^ | .07 |  | .04 | .16^*^ | .00 | .07 | .15^*^ | .04 |
| I believe that attending religious services (masses, weddings, funerals, etc.) is important. | .26^*^ | .21^*^ | .20^*^ | .21^*^ | .31^*^ | .20^*^ |  | .10 | .16^*^ | .20^*^ | .13^*^ | .29^*^ | .20^*^ |
| I prefer to believe what is said on social networks or on the Internet rather than what science says. | -.21^*^ | -.04 | -.03 | -.08 | -.22^*^ | -.27^*^ |  | -.19^*^ | -.05 | -.10 | -.18^*^ | -.20^*^ | -.26^*^ |
| I play a musical instrument, or I studied singing. | -.05 | .07 | -.02 | -.03 | .06 | .07 |  | .03 | .09 | .09 | .01 | .12 | .10 |
| I love animals. | .10 | .03 | .03 | .02 | .03 | .07 |  | -.01 | .02 | .03 | .04 | -.12 | .00 |
| I prefer science to art. | -.06 | -.07 | .13^*^ | .09 | .12^*^ | .07 |  | .08 | -.10 | .14^*^ | .14^*^ | .09 | .01 |
| I listen to music. | -.01 | .03 | .09 | -.10 | .01 | .13^*^ |  | -.04 | .06 | -.10 | -.09 | .00 | .08 |
| I write or I wrote a personal diary. | -.15^*^ | .11 | -.13^*^ | -.18^*^ | .00 | .17^*^ |  | -.07 | .13^*^ | -.14^*^ | -.15^*^ | .03 | .16^*^ |
| I happened to write poems, stories, or books. | -.09 | -.02 | -.14^*^ | -.14^*^ | .12^*^ | .30^*^ |  | .04 | .08 | -.01 | .00 | .23^*^ | .36^*^ |
| I play sports. | -.02 | -.18^*^ | .33^*^ | .09 | .12^*^ | .08 |  | -.09 | -.19^*^ | .18^*^ | .06 | .16^*^ | .12 |
| I was volunteering. | -.05 | .01 | -.06 | -.02 | .08 | .09 |  | -.09 | -.02 | .05 | -.05 | .04 | .10 |
| I feel good. | .08 | -.12^*^ | .55^*^ | .26^*^ | .13^*^ | .02 |  | .08 | -.06 | .23^*^ | .12 | .10 | .06 |
| I make jokes or laugh at other people's jokes. | -.05 | -.01 | .31^*^ | .03 | .01 | .01 |  | -.06 | .02 | -.03 | -.12 | -.09 | -.01 |
| I am or I was seriously engaged. | -.16^*^ | -.17^*^ | -.02 | -.01 | -.15^*^ | -.14^*^ |  | -.10 | -.08 | .05 | .01 | -.03 | -.02 |
| When there is no school, I sleep late regardless of when I go to bed. | -.06 | -.03 | .00 | -.14^*^ | -.19^*^ | -.11 |  | -.08 | .02 | -.05 | -.13^*^ | -.20^*^ | -.09 |
| I listen to older people with respect. | .35^*^ | .19^*^ | .27^*^ | .37^*^ | .33^*^ | .18^*^ |  | .29^*^ | .13^*^ | .18^*^ | .27^*^ | .32^*^ | .18^*^ |
| I say 'please' and 'thank you'. | .32^*^ | .24^*^ | .12^*^ | .26^*^ | .33^*^ | .24^*^ |  | .20^*^ | .05 | .13^*^ | .17^*^ | .26^*^ | .22^*^ |
| I try to keep the promises I made. | .26^*^ | .05 | .21^*^ | .27^*^ | .40^*^ | .25^*^ |  | .07 | -.01 | .13^*^ | .06 | .33^*^ | .22^*^ |

^*^ FDR corrected p<.05 within each table.

Note. Criteria are presented only in self-report form. In the observer form, they were presented in third person with the subject ‘he/she’.

**Table E.** Correlation matrix between the six HEXACO-MSI-E traits and (a) the self-report (*N* = 382) and (b) the reported by an observer questions regarding job (*N* = 373)

|  | **(a) Self-Report Criteria** | | | | | | | **(b) Observer Criteria** | | | | | |
| --- | --- | --- | --- | --- | --- | --- | --- | --- | --- | --- | --- | --- | --- |
|  | **HEXACO-MSI-E** | | | | | | | **HEXACO-MSI-E** | | | | | |
| **Questions regarding a possible future job** | **H** | **E** | **X** | **A** | **C** | **O** |  | **H** | **E** | **X** | **A** | **C** | **O** |
| It is important for you that the job is honest. | .25^*^ | .13^*^ | .06 | .07 | .15^*^ | .12^*^ |  | .17^*^ | .03 | .04 | .09 | .02 | .09 |
| It is important for you that the job guarantees a good income. | -.21^*^ | -.11 | .06 | -.13^*^ | -.10 | -07 |  | -.23^*^ | -.10 | .12 | -.09 | -.03 | -.04 |
| It is important for you that the job provides emotional security. | .04 | .09 | .00 | .05 | .06 | .13^*^ |  | .04 | .08 | .04 | .05 | .04 | .04 |
| It is important for you that the job allows you collaborate with other people. | .10 | .10 | .19^*^ | .14^*^ | .12^*^ | .06 |  | .08 | .06 | .10 | .13 | .06 | .08 |
| It is important for you that the job is organized and planned. | .10 | .07 | .19^*^ | .07 | .19^*^ | .07 |  | -.01 | -.05 | .12 | .07 | .13 | .06 |
| It is important for you that your job allows you to be in contact with nature. | .20^*^ | .13^*^ | -.03 | .20^*^ | .19^*^ | .19^*^ |  | .10 | .08 | .05 | .11 | .06 | .14 |
| It is important for you that the job allows you to be in contact with art. | .12^*^ | .09 | -.04 | .02 | .18^*^ | .33^*^ |  | .07 | .07 | -.07 | .06 | .06 | .24^*^ |

^*^ FDR corrected p<.05 within each table.

Note. Criteria are presented only in self-report form. In the observer form, they were presented in third person with the subject ‘your son/daughter’.

**Table F.** Frequency distributions and *F* value for (a) the self-report (*N* = 388) and (b) the reported by an observer (*N* = 373) Internalizing scale of youth emotional and behavioral problems

|  | **Youth Emotional and Behavioral Problems – Internalizing scale** | | | | |
| --- | --- | --- | --- | --- | --- |
|  | **Self-Report (YSR)** | |  | **Observer (YOR)** | |
| **Scores** | **Frequency** | **Percent** |  | **Frequency** | **Percent** |
| 20.00 | 9 | 2.3 |  | 13 | 3.5 |
| 21.00 | 10 | 2.6 |  | 15 | 4.0 |
| 22.00 | 18 | 4.6 |  | 19 | 5.1 |
| 23.00 | 14 | 3.6 |  | 25 | 6.7 |
| 24.00 | 15 | 3.9 |  | 25 | 6.7 |
| 25.00 | 19 | 4.9 |  | 22 | 5.9 |
| 26.00 | 14 | 3.6 |  | 21 | 5.6 |
| 27.00 | 19 | 4.9 |  | 24 | 6.4 |
| 28.00 | 18 | 4.6 |  | 23 | 6.2 |
| 29.00 | 17 | 4.4 |  | 33 | 8.8 |
| 30.00 | 27 | 7.0 |  | 25 | 6.7 |
| 31.00 | 14 | 3.6 |  | 14 | 3.8 |
| 32.00 | 17 | 4.4 |  | 14 | 3.8 |
| 33.00 | 10 | 2.6 |  | 17 | 4.6 |
| 34.00 | 15 | 3.9 |  | 19 | 5.1 |
| 35.00 | 18 | 4.6 |  | 9 | 2.4 |
| 36.00 | 14 | 3.6 |  | 16 | 4.3 |
| 37.00 | 15 | 3.9 |  | 8 | 2.1 |
| 38.00 | 12 | 3.1 |  | 5 | 1.3 |
| 39.00 | 6 | 1.5 |  | 6 | 1.6 |
| 40.00 | 5 | 1.3 |  | 3 | .8 |
| 41.00 | 7 | 1.8 |  | 5 | 1.3 |
| 42.00 | 9 | 2.3 |  | 4 | 1.1 |
| 43.00 | 7 | 1.8 |  | 4 | 1.1 |
| 44.00 | 9 | 2.3 |  | - | - |
| 45.00 | 12 | 3.1 |  | 1 | .3 |
| 46.00 | 4 | 1.0 |  | - | - |
| 47.00 | 5 | 1.3 |  | 1 | .3 |
| 48.00 | 3 | .8 |  | - | - |
| 49.00 | 7 | 1.8 |  | - | - |
| 50.00 | 2 | .5 |  | - | - |
| 51.00 | 1 | .3 |  | - | - |
| 52.00 | 3 | .8 |  | - | - |
| 53.00 | 2 | .5 |  | 1 | .3 |
| 54.00 | 6 | 1.5 |  | 1 | .3 |
| 55.00 | 1 | .3 |  | - | - |
| 56.00 | 2 | .5 |  | - | - |
| 58.00 | 2 | .5 |  | - | - |
| Total | 388 | 100.0 |  | 373 | 100.0 |
| Model | *F*(5,382)=138.20^***^ | |  | *F*(1,371)=104.31^***^ | |

*Note.* *** p < .001.

**Table G.** Frequency distributions and *F* value for (a) the self-report (*N* = 388) and (b) the reported by an observer (*N* = 373) Externalizing scale of youth emotional and behavioral problems

|  | **Youth Emotional and Behavioral Problems – Externalizing scale** | | | | |
| --- | --- | --- | --- | --- | --- |
|  | **Self-Report (YSR)** | |  | **Observer (YOR)** | |
| **Scores** | **Frequency** | **Percent** |  | **Frequency** | **Percent** |
| 27.00 | 8 | 2.1 |  | 18 | 4.8 |
| 28.00 | 9 | 2.3 |  | 25 | 6.7 |
| 29.00 | 20 | 5.2 |  | 35 | 9.4 |
| 30.00 | 21 | 5.4 |  | 32 | 8.6 |
| 31.00 | 24 | 6.2 |  | 32 | 8.6 |
| 32.00 | 29 | 7.5 |  | 34 | 9.1 |
| 33.00 | 28 | 7.2 |  | 44 | 11.8 |
| 34.00 | 23 | 5.9 |  | 25 | 6.7 |
| 35.00 | 16 | 4.1 |  | 25 | 6.7 |
| 36.00 | 22 | 5.7 |  | 25 | 6.7 |
| 37.00 | 25 | 6.4 |  | 14 | 3.8 |
| 38.00 | 18 | 4.6 |  | 8 | 2.1 |
| 39.00 | 18 | 4.6 |  | 13 | 3.5 |
| 40.00 | 14 | 3.6 |  | 7 | 1.9 |
| 41.00 | 17 | 4.4 |  | 3 | .8 |
| 42.00 | 12 | 3.1 |  | 7 | 1.9 |
| 43.00 | 6 | 1.5 |  | 8 | 2.1 |
| 44.00 | 8 | 2.1 |  | 6 | 1.6 |
| 45.00 | 8 | 2.1 |  | 1 | .3 |
| 46.00 | 8 | 2.1 |  | 3 | .8 |
| 47.00 | 8 | 2.1 |  | 1 | .3 |
| 48.00 | 4 | 1.0 |  | 3 | .8 |
| 49.00 | 7 | 1.8 |  | - | - |
| 50.00 | - | - |  | 1 | .3 |
| 51.00 | 3 | .8 |  | 3 | .8 |
| 52.00 | 8 | 2.1 |  | - | - |
| 53.00 | 5 | 1.3 |  | - | - |
| 54.00 | 4 | 1.0 |  | - | - |
| 55.00 | 1 | .3 |  | - | - |
| 57.00 | 6 | 1.5 |  | - | - |
| 58.00 | 3 | .8 |  | - | - |
| 59.00 | 2 | .5 |  | - | - |
| 63.00 | 1 | .3 |  | - | - |
| 64.00 | 1 | .3 |  | - | - |
| 71.00 | 1 | .3 |  | - | - |
| Total | 388 | 100.0 |  | 373 | 100.0 |
| Model | *F*(4,383)=119.30^***^ | |  | *F*(3,369)=23.70^***^ | |

*Note.* *** p < .001.

**Table H.** Frequency distributions and *F* value of (a) the self-report (*N* = 388) and (b) the reported by an observer (*N* = 373) Anxious/Depressed scale of youth emotional and behavioral problems

|  | **Youth Emotional and Behavioral Problems – Anxious/Depressed scale** | | | | |
| --- | --- | --- | --- | --- | --- |
|  | **Self-Report (YSR)** | |  | **Observer (YOR)** | |
| **Scores** | **Frequency** | **Percent** |  | **Frequency** | **Percent** |
| 10.00 | 19 | 4.9 |  | 23 | 6.2 |
| 11.00 | 30 | 7.7 |  | 34 | 9.1 |
| 12.00 | 27 | 7.0 |  | 37 | 9.9 |
| 13.00 | 28 | 7.2 |  | 44 | 11.8 |
| 14.00 | 23 | 5.9 |  | 35 | 9.4 |
| 15.00 | 37 | 9.5 |  | 38 | 10.2 |
| 16.00 | 29 | 7.5 |  | 49 | 13.1 |
| 17.00 | 29 | 7.5 |  | 26 | 7.0 |
| 18.00 | 32 | 8.2 |  | 29 | 7.8 |
| 19.00 | 26 | 6.7 |  | 20 | 5.4 |
| 20.00 | 16 | 4.1 |  | 13 | 3.5 |
| 21.00 | 18 | 4.6 |  | 9 | 2.4 |
| 22.00 | 8 | 2.1 |  | 3 | .8 |
| 23.00 | 14 | 3.6 |  | 6 | 1.6 |
| 24.00 | 13 | 3.4 |  | 4 | 1.1 |
| 25.00 | 11 | 2.8 |  | - | - |
| 26.00 | 9 | 2.3 |  | 1 | .3 |
| 27.00 | 8 | 2.1 |  | - | - |
| 28.00 | 7 | 1.8 |  | 1 | .3 |
| 29.00 | 3 | .8 |  | 1 | .3 |
| 30.00 | 1 | .3 |  | - | - |
| Total | 388 | 100.0 |  | 373 | 100.0 |
| Model | *F*(5,382)=110.37^***^ | |  | *F*(1,371)=66.35^***^ | |

*Note.* *** p < .001.

**Table I.** Frequency distributions and *F* value for (a) the self-report (*N* = 388) and (b) the reported by an observer (*N* = 373) Withdrawn/Depressed scale of youth emotional and behavioral problems

|  | **Youth Emotional and Behavioral Problems – Withdrawn/Depressed scale** | | | | |
| --- | --- | --- | --- | --- | --- |
|  | **Self-Report (YSR)** | |  | **Observer (YOR)** | |
| **Scores** | **Frequency** | **Percent** |  | **Frequency** | **Percent** |
| 7.00 | 44 | 11.3 |  | 48 | 12.9 |
| 8.00 | 50 | 12.9 |  | 61 | 16.4 |
| 9.00 | 58 | 14.9 |  | 70 | 18.8 |
| 10.00 | 37 | 9.5 |  | 49 | 13.1 |
| 11.00 | 42 | 10.8 |  | 39 | 10.5 |
| 12.00 | 43 | 11.1 |  | 34 | 9.1 |
| 13.00 | 27 | 7.0 |  | 27 | 7.2 |
| 14.00 | 19 | 4.9 |  | 15 | 4.0 |
| 15.00 | 20 | 5.2 |  | 13 | 3.5 |
| 16.00 | 9 | 2.3 |  | 4 | 1.1 |
| 17.00 | 11 | 2.8 |  | 5 | 1.3 |
| 18.00 | 11 | 2.8 |  | 5 | 1.3 |
| 19.00 | 10 | 2.6 |  | 1 | .3 |
| 20.00 | 2 | .5 |  | 2 | .5 |
| 21.00 | 5 | 1.3 |  | - | - |
| Total | 388 | 100.0 |  | 373 | 100.0 |
| Model | *F*(3,384)=189.95^***^ | |  | *F*(3,369)=35.32^***^ | |

*Note.* *** p < .001.

**Table J.** Frequency distributions and *F* value for (a) the self-report (*N* = 388) and (b) the reported by an observer (*N* = 373) Somatic Complaints scale of youth emotional and behavioral problems

|  | **Youth Emotional and Behavioral Problems – Somatic Complaints scale** | | | | |
| --- | --- | --- | --- | --- | --- |
|  | **Self-Report (YSR)** | |  | **Observer (YOR)** | |
| **Scores** | **Frequency** | **Percent** |  | **Frequency** | **Percent** |
| 3.00 | 103 | 26.5 |  | 211 | 56.6 |
| 4.00 | 100 | 25.8 |  | 108 | 29.0 |
| 5.00 | 76 | 19.6 |  | 40 | 10.7 |
| 6.00 | 44 | 11.3 |  | 11 | 2.9 |
| 7.00 | 43 | 11.1 |  | 3 | .8 |
| 8.00 | 17 | 4.4 |  | - | - |
| 9.00 | 5 | 1.3 |  | - | - |
| Total | 388 | 100.0 |  | 373 | 100.0 |
| Model | *F*(3,384)=47.27^***^ | |  | *F*(1,371)=28.15^***^ | |

*Note.* *** p < .001.

**Table K.** Frequency distributions and *F* value for (a) the self-report (*N* = 388) and (b) the reported by an observer (*N* = 373) Delinquent Behavior scale of youth emotional and behavioral problems

|  | **Youth Emotional and Behavioral Problems – Delinquent Behavior scale** | | | | |
| --- | --- | --- | --- | --- | --- |
|  | **Self-Report (YSR)** | |  | **Observer (YOR)** | |
| **Scores** | **Frequency** | **Percent** |  | **Frequency** | **Percent** |
| 11.00 | 76 | 19.6 |  | 134 | 35.9 |
| 12.00 | 62 | 16.0 |  | 105 | 28.2 |
| 13.00 | 71 | 18.3 |  | 80 | 21.4 |
| 14.00 | 57 | 14.7 |  | 26 | 7.0 |
| 15.00 | 43 | 11.1 |  | 18 | 4.8 |
| 16.00 | 19 | 4.9 |  | 7 | 1.9 |
| 17.00 | 12 | 3.1 |  | 1 | .3 |
| 18.00 | 15 | 3.9 |  | 1 | .3 |
| 19.00 | 9 | 2.3 |  | 1 | .3 |
| 20.00 | 8 | 2.1 |  | - | - |
| 21.00 | 4 | 1.0 |  | - | - |
| 22.00 | 3 | .8 |  | - | - |
| 23.00 | 4 | 1.0 |  | - | - |
| 24.00 | 3 | .8 |  | - | - |
| 27.00 | 2 | .5 |  | - | - |
| Total | 388 | 100.0 |  | 373 | 100.0 |
| Model | *F*(4,383)=57.61^***^ | |  | *F*(2,370)=10.97^***^ | |

*Note.* *** p < .001.

**Table L.** Frequency distributions and *F* value for (a) the self-report (*N* = 388) and (b) the reported by an observer (*N* = 373) Aggressive Behavior scale of youth emotional and behavioral problems

|  | **Youth Emotional and Behavioral Problems – Aggressive Behavior scale** | | | | |
| --- | --- | --- | --- | --- | --- |
|  | 1. **Self-Report (YSR)** | |  | 1. **Observer (YOR)** | |
| **Scores** | **Frequency** | **Percent** |  | **Frequency** | **Percent** |
| 16.00 | 17 | 4.4 |  | 26 | 7.0 |
| 17.00 | 25 | 6.4 |  | 30 | 8.0 |
| 18.00 | 19 | 4.9 |  | 40 | 10.7 |
| 19.00 | 29 | 7.5 |  | 50 | 13.4 |
| 20.00 | 35 | 9.0 |  | 37 | 9.9 |
| 21.00 | 30 | 7.7 |  | 40 | 10.7 |
| 22.00 | 23 | 5.9 |  | 33 | 8.8 |
| 23.00 | 31 | 8.0 |  | 26 | 7.0 |
| 24.00 | 30 | 7.7 |  | 25 | 6.7 |
| 25.00 | 23 | 5.9 |  | 12 | 3.2 |
| 26.00 | 25 | 6.4 |  | 10 | 2.7 |
| 27.00 | 14 | 3.6 |  | 10 | 2.7 |
| 28.00 | 15 | 3.9 |  | 9 | 2.4 |
| 29.00 | 11 | 2.8 |  | 6 | 1.6 |
| 30.00 | 10 | 2.6 |  | 9 | 2.4 |
| 31.00 | 11 | 2.8 |  | 2 | .5 |
| 32.00 | 9 | 2.3 |  | 3 | .8 |
| 33.00 | 4 | 1.0 |  | 2 | .5 |
| 34.00 | 9 | 2.3 |  | - | - |
| 35.00 | 7 | 1.8 |  | 3 | .8 |
| 36.00 | 2 | .5 |  | - | - |
| 37.00 | 2 | .5 |  | - | - |
| 38.00 | 2 | .5 |  | - | - |
| 39.00 | 1 | .3 |  | - | - |
| 40.00 | 1 | .3 |  | - | - |
| 41.00 | 1 | .3 |  | - | - |
| 44.00 | 2 | .5 |  | - | - |
| Total | 388 | 100.0 |  | 373 | 100.0 |
| Model | *F*(4,383)=120.76^***^ | |  | *F*(3,369)=27.69^***^ | |

*Note.* *** p < .001.

**Table M.** Frequency distributions (percent) and *F* value for (a) the self-report (*N* = 385 if not otherwise indicated) and (b) the reported by an observer youth risk behaviors (*N* = 373 if not otherwise indicated)

|  | **(a) Self-Report Criteria** | | | | | | | |  |  | **(b) Observer Criteria** | | | | | | | | |
| --- | --- | --- | --- | --- | --- | --- | --- | --- | --- | --- | --- | --- | --- | --- | --- | --- | --- | --- | --- |
| **Youth Risk Behaviors** | **Model** | **0** | **1^e^** |  |  |  |  |  |  |  | **Model** | **0** | **1^e^** |  |  |  |  |  |  |
| Have you ever ridden in a car driven by someone who had been drinking alcohol? | *F*(1,383)=8.54^**^ | 91.7 | 8.3 |  |  |  |  |  |  |  | - | 98.1 | 1.9 |  |  |  |  |  |  |
| Have you ever carried a weapon, such as a gun, knife, or club? | *F*(1,383)=27.84^***^ | 94.3 | 5.7 |  |  |  |  |  |  |  | - | 99.2 | .8 |  |  |  |  |  |  |
| Apart from a few sips, have you ever had a whole alcoholic drink? | *F*(1,383)=35.12^***^ | 90.4 | 9.6 |  |  |  |  |  |  |  | *F*(1,371)=3.96^*^ | 97.6 | 2.4 |  |  |  |  |  |  |
| Have you ever used marijuana for a relatively continuous period? | *F*(1,383)=6.32^*^ | 98.7 | 1.3 |  |  |  |  |  |  |  | - | - | - |  |  |  |  |  |  |
| Have you ever engaged in self-harming behaviors (cutting yourself, scratching yourself, etc.) on a voluntary basis? | *F*(2,382)=16.07^***^ | 87.0 | 13.0 |  |  |  |  |  |  |  | *F*(1,371)=13.22^***^ | 97.6 | 2.4 |  |  |  |  |  |  |
| Have you ever been involved in a physical fight (e.g., you got into a fight, brawls, etc.)? | *F*(2,382)=25.38^***^ | 72.5 | 27.5 |  |  |  |  |  |  |  | *F*(1,371)=4.82^*^ | 96.5 | 3.5 |  |  |  |  |  |  |
| Have you ever been bullied at school? | *F*(1,383)=21.61^***^ | 75.8 | 24.2 |  |  |  |  |  |  |  | *F*(1,371)=18.53^***^ | 84.2 | 15.8 |  |  |  |  |  |  |
| Have you ever been electronically bullied (through texting, Instagram, Facebook, or other social media)? | *F*(3,381)=11.57^***^ | 88.8 | 11.2 |  |  |  |  |  |  |  | *F*(4,368)=8.36^***^ | 92.8 | 7.2 |  |  |  |  |  |  |
|  | **Model** | **1^f^** | **2^f^** | **3^f^** | **4^f^** | **5^f^** |  |  |  |  | **Model** | **1^f^** | **2^f^** | **3^f^** | **4^f^** | **5^f^** |  |  |  |
| When you ride a bike, skateboard or overboard (etc.), how often do you wear a helmet?^a, c^ | *F*(2,303)=49.18^***^ | 47.7 | 21.6 | 9.2 | 11.4 | 10.1 |  |  |  |  | *F*(1,239)=14.71^***^ | 53.1 | 12.4 | 12.0 | 10.0 | 12.4 |  |  |  |
|  | **Model** | **1^g^** | **2^g^** | **3^g^** | **4^g^** | **5^g^** |  |  |  |  | **Model** | **1^g^** | **2^g^** | **3^g^** | **4^g^** | **5^g^** |  |  |  |
| How often do you wear a seat belt when riding in a car? | *F*(1,383)=36.03^***^ | 9.1 | 17.4 | 20.5 | 22.9 | 30.1 |  |  |  |  | *F*(1,371)=6.97^**^ | 9.9 | 14.7 | 16.9 | 21.2 | 37.3 |  |  |  |
|  | **Model** | **1^h^** | **2^h^** | **3^h^** | **4^h^** | **5^h^** |  |  |  |  | **Model** | **1^h^** | **2^h^** | **3^h^** | **4^h^** | **5^h^** |  |  |  |
| How old were you when you first tried smoking a cigarette, even just one or two puffs? | *F*(2,382)=8.33^***^ | 0 | 2.3 | 2.3 | 1.3 | 94.0 |  |  |  |  | - | - | - | - | - | - | - |  |  |
|  | **Model** | **1^i^** | **2^i^** | **3^i^** | **4^i^** | **5^i^** | **6^i^** |  |  |  | **Model** | **1^i^** | **2^i^** | **3^i^** | **4^i^** | **5^i^** | **6^i^** |  |  |
| In the last 30 days, on the days you smoked, how many cigarettes (regular or electronic) per day have you smoked? | - | 98.7 | .8 | .3 | .3 | 0 | 0 |  |  |  | - | 100.0 | 0 | 0 | 0 | 0 | 0 |  |  |
|  | **Model** | **1^j^** | **2^j^** | **3^j^** | **4^j^** | **5^j^** | **6^j^** | **7^j^** | **8^j^** |  | **Model** | **1^j^** | **2^j^** | **3^j^** | **4^j^** | **5^j^** | **6^j^** | **7^j^** | **8^j^** |
| During the past 7 days, how many days have you eaten breakfast? | *F*(3,381)=8.47^***^ | 11.4 | 3.1 | 6.2 | 8.6 | 7.5 | 7.8 | 6.8 | 48.6 |  | *F*(1,371)=7.80^**^ | 8.0 | 2.9 | 4.0 | 4.6 | 3.8 | 5.6 | 6.7 | 64.3 |
|  | **Model** | **1^k^** | **2^k^** | **3^k^** | **4^k^** | **5^k^** | **6^k^** | **7^k^** |  |  | **Model** | **1^k^** | **2^k^** | **3^k^** | **4^k^** | **5^k^** | **6^k^** | **7^k^** |  |
| When you have class, how many hours do you sleep per night? | *F*(3,381)=14.32^***^ | 2.9 | 4.9 | 11.2 | 27.0 | 36.6 | 12.7 | 4.7 |  |  | *F*(1,371)=32.04^***^ | .3 | 1.1 | 5.9 | 19.8 | 47.7 | 21.4 | 3.8 |  |
|  | **Model** | **1^l^** | **2^l^** | **3^l^** | **4^l^** | **5^l^** | **6^l^** |  |  |  | **Model** | **1^l^** | **2^l^** | **3^l^** | **4^l^** | **5^l^** | **6^l^** |  |  |
| On a typical school day (even if in distance learning), how many hours a day do you watch TV?^b, d^ | *F*(1,297)=11.91^***^ | 30.1 | 16.7 | 24.1 | 16.4 | 4.7 | 8.0 |  |  |  | - | 21.2 | 26.8 | 31.8 | 14.0 | 4.0 | 2.2 |  |  |
|  | **Model** | **1^m^** | **2^m^** | **3^m^** | **4^m^** | **5^m^** | **6^m^** | **7^m^** |  |  | **Model** | **1^m^** | **2^m^** | **3^m^** | **4^m^** | **5^m^** | **6^m^** | **7^m^** |  |
| On a typical school day (even if in distance learning), on average, how many hours a day do you play video games or use computer or mobile phone for things not related to school? | *F*(2,382)=11.55^***^ | 10.6 | 5.7 | 11.9 | 16.1 | 16.9 | 15.1 | 23.6 |  |  | *F*(2,370)=15.40^***^ | 4.0 | 3.8 | 11.0 | 27.1 | 20.4 | 13.9 | 19.8 |  |

*Note 1*. For Self-Report criteria: ^a^n = 306; ^b^n = 299. For Observer criteria: ^c^n = 241; ^d^n = 321.

*Note 2.* * p < .05; ** < .01; *** p < .001.

*Note 3.* 1^e^ = *Yes*; 1^f^ = *I never wear a helmet*; 2**^f^** = *I rarely wear a helmet*; 3^f^ = *I sometimes wear a helmet*; 4^f^ = *Most of the time I wear a helmet*; 5^f^ = *I always wear a helmet*; 1^g^ = *Never*; 2^g^ = *Rarely*; 3^g^ = *Sometimes*; 4^g^ = *Almost always*; 5^g^ = *Always*; 1^h^ = *8 years or younger*; 2^h^ = *9-10 years old*; 3^h^ = *11-12 years old*; 4^h^ = *13 years or older*; 5^h^ = *I have never tried smoking a cigarette, not even one or two puffs*; 1^i^ = *I have not smoked cigarettes in the last 30 days*; 2^i^ = *1 cigarette per day*; 3^i^ = *2 to 5 cigarettes per day*; 4^i^ = *6 to 10 cigarettes per day*; 5^i^ = *11 to 20 cigarettes per day*; 6^i^ = *More than 20 cigarettes per day*; 1^j^ = *0 days*; 2^j^ = *1 day*; 3^j^ = *2 days*; 4^j^ = *3 days*; 5^j^ = *4 days*; 6^j^ = *5 days*; 7^j^ = *6 days*; 8^j^ = *7 days*; 1^k^ = *4 hours or less*; 2^k^ = *5 hours*; 3^k^ = *6 hours*; 4^k^ = *7 hours*; 5^k^ = *8 hours*; 6^k^ = *9 hours*; 7^k^ = *10 or more hours*; 1^l^ = *Less than 1 hour per day*; 2^l^ = *1 hour per day*; 3^l^ = *2 hours per day*; 4^l^ = *3 hours per day*; 5^l^ = *4 hours per day*; 6^l^ = *5 or more hours per day*; 1^m^ = *None*; 2^m^ = *Less than 1 hour per day*; 3^m^ = *1 hour per day*; 4^m^ = *2 hours per day*; 5^m^ = *3 hours per day*; 6^m^ = *4 hours per day*; 7^m^ = *5 or more hours per day*.

*Note 4.* Criteria are presented only in self-report form. In the observer form, they were presented in third person with the subject ‘your son/daughter’.

**Table N.** Frequency distributions (percent) and *F* value for (a) the self-report (*N* = 382) and (b) the reported by an observer values, beliefs, and behaviors (*N* = 373)

|  | **(a) Self-Report Criteria** | | | | | | | **(b) Observer Criteria** | | | | | |
| --- | --- | --- | --- | --- | --- | --- | --- | --- | --- | --- | --- | --- | --- |
| **Adolescents’ values, beliefs, and behaviors** | **Model** | **0** | **1^a^** |  |  |  |  | **Model** | **0** | **1^a^** |  |  |  |
| I believe in God. | *F*(3,378)=8.67^***^ | 15.7 | 84.3 |  |  |  |  | *F*(2,370)=8.00^***^ | 5.1 | 94.9 |  |  |  |
| I play a musical instrument, or I studied singing. | - | 46.9 | 53.1 |  |  |  |  | *F*(1,371)=5.33^*^ | 53.9 | 46.1 |  |  |  |
| I am or I was seriously engaged. | *F*(2,379)=9.01^***^ | 80.6 | 19.4 |  |  |  |  | - | 97.6 | 2.4 |  |  |  |
|  | **Model** | **1^b^** | **2^b^** | **3^b^** | **4^b^** | **5^b^** |  | **Model** | **1^b^** | **2^b^** | **3^b^** | **4^b^** | **5^b^** |
| I believe that attending religious services (masses, weddings, funerals, etc.) is important. | *F*(4,377)=19.41^***^ | 10.5 | 12.6 | 12.8 | 30.4 | 33.8 |  | *F*(3,369)=16.26^***^ | 5.6 | 16.4 | 21.2 | 35.9 | 20.9 |
| I prefer to believe what is said on social networks or on the Internet rather than what science says. | *F*(2,379)=21.91^***^ | 50.0 | 25.9 | 12.0 | 7.9 | 4.2 |  | *F*(2,370)=18.72^***^ | 44.0 | 28.7 | 18.5 | 7.0 | 1.9 |
| I love animals. | *F*(1,380)=4.09^*^ | 3.1 | 4.5 | 7.1 | 15.2 | 70.2 |  | *F*(1,371)=5.57^*^ | 1.1 | 4.6 | 6.4 | 24.4 | 63.5 |
|  | **Model** | **1^c^** | **2^c^** | **3^c^** | **4^c^** | **5^c^** |  | **Model** | **1^c^** | **2^c^** | **3^c^** | **4^c^** | **5^c^** |
| I prefer science to art. | *F*(1,380)=6.74^**^ | 14.1 | 18.1 | 21.7 | 14.9 | 31.2 |  | *F*(2,370)=6.18^**^ | 11.3 | 19.8 | 27.9 | 20.6 | 20.4 |
| I listen to music. | *F*(2,379)=6.14^**^ | 1.8 | 7.1 | 9.4 | 25.7 | 56.0 |  | - | 1.1 | 4.6 | 14.7 | 39.4 | 40.2 |
| I write or I wrote a personal diary. | *F*(5,376)=9.39^***^ | 48.7 | 15.2 | 17.3 | 7.6 | 11.3 |  | *F*(3,369)=10.21^***^ | 51.7 | 13.1 | 22.5 | 8.3 | 4.3 |
| I happened to write poems, stories, or books. | *F*(3,378)=22.27^***^ | 52.1 | 13.9 | 19.4 | 8.9 | 5.8 |  | *F*(1,371)=56.42^***^ | 48.8 | 19.8 | 21.7 | 8.0 | 1.6 |
| I play sports. | *F*(2,379)=26.84^***^ | 13.6 | 13.9 | 19.1 | 24.3 | 29.1 |  | *F*(3,369)=11.09^***^ | 12.6 | 19.6 | 22.0 | 22.8 | 23.1 |
| I was volunteering. | - | 66.0 | 9.7 | 13.1 | 7.1 | 4.2 |  | - | 82.8 | 9.4 | 5.1 | 1.6 | 1.1 |
| I feel good. | *F*(3,378)=60.73^***^ | 2.4 | 5.2 | 18.8 | 29.1 | 44.5 |  | *F*(1,371)=21.22^***^ | 0.8 | 0.5 | 4.8 | 29.2 | 64.6 |
| I make jokes or laugh at other people's jokes. | *F*(1,380)=39.42^***^ | 4.5 | 6.0 | 12.3 | 36.6 | 40.6 |  | *F*(1,371)=4.97^*^ | 5.4 | 2.1 | 13.4 | 38.3 | 40.8 |
| When there is no school, I sleep late regardless of when I go to bed. | *F*(1,380)=14.24^***^ | 9.4 | 10.2 | 19.6 | 28.0 | 32.7 |  | *F*(1,371)=14.98^***^ | 12.1 | 13.9 | 29.8 | 268 | 17.4 |
| I listen to older people with respect. | *F*(5,376)=25.14^***^ | 2.6 | 5.5 | 20.2 | 31.9 | 39.8 |  | *F*(2,370)=30.40^***^ | .5 | 3.5 | 8.6 | 29.2 | 58.2 |
| I say 'please' and 'thank you'. | *F*(3,378)=26.92^***^ | 2.4 | 3.9 | 7.9 | 24.9 | 61.0 |  | *F*(3,369)=12.69^***^ | .8 | 3.2 | 10.2 | 31.4 | 54.4 |
| I try to keep the promises I made. | *F*(2,379)=41.95^***^ | 1.3 | 3.9 | 18.3 | 35.9 | 40.6 |  | *F*(1,371)=44.35^***^ | .8 | 3.8 | 25.5 | 42.9 | 27.1 |

*Note 1.* * p < .05; ** < .01; *** p < .001.

*Note 2*. 0 = *No*; 1^a^ = *Yes*; 1^b^ = *Not at all*; 2^b^ = *A little*; 3^b^ = *Moderately*; 4^b^ = *Quite a bit*; 5^b^ = *Very much*; 1^c^ = *Never*; 2^c^ = *A little*; 3^c^ = *Sometimes*; 4^c^ = *Often*; 5^c^ = *Always*.

*Note 3.* Criteria are presented only in self-report form. In the observer form, they were presented in third person with the subject ‘he/she’.

**Table O.** Frequency distributions (percent) and *F* value for (a) the self-report (*N* = 382) and (b) the reported by an observer questions regarding job (*N* = 373)

|  | **(a) Self-Report Criteria** | | | | | | **(b) Observer Criteria** | | | | | | |
| --- | --- | --- | --- | --- | --- | --- | --- | --- | --- | --- | --- | --- | --- |
| **Questions regarding a possible future job** | **Model** | **1** | **2** | **3** | **4** | **5** |  | **Model** | **1** | **2** | **3** | **4** | **5** |
| It is important for you that the job is honest. | *F*(1,380)=25.35^***^ | 1.8 | 1.0 | 17.0 | 29.6 | 50.5 |  | *F*(1,371)=11.45^***^ | .0 | 0.8 | 18.8 | 28.2 | 52.3 |
| It is important for you that the job guarantees a good income. | *F*(1,380)=16.97^***^ | 1.0 | 6.3 | 41.4 | 28.3 | 23.0 |  | *F*(2,370)=15.16^***^ | .5 | 3.8 | 34.6 | 37.3 | 23.9 |
| It is important for you that the job provides emotional security. | *F*(1,380)=7.00^**^ | 1.3 | 3.9 | 30.4 | 37.2 | 27.2 |  | - | .3 | 4.3 | 29.2 | 38.6 | 27.6 |
| It is important for you that the job allows you collaborate with other people. | *F*(2,379)=10.74^***^ | 3.9 | 7.1 | 31.4 | 33.5 | 24.1 |  | *F*(1,371)=6.28^*^ | 2.1 | 7.0 | 37.3 | 33.0 | 20.6 |
| It is important for you that the job is organized and planned. | *F*(2,379)=11.76^***^ | 2.4 | 7.1 | 32.2 | 29.8 | 28.5 |  | *F*(1,371)=6.22^*^ | 1.9 | 8.3 | 40.2 | 33.2 | 16.4 |
| It is important for you that your job allows you to be in contact with nature. | *F*(3,378)=10.45^***^ | 9.7 | 29.8 | 34.3 | 15.2 | 11.0 |  | *F*(1,371)=7.42^**^ | 8.3 | 34.9 | 27.6 | 18.0 | 11.3 |
| It is important for you that the job allows you to be in contact with art. | *F*(2,379)=25.96^***^ | 24.1 | 30.4 | 27.5 | 9.7 | 8.4 |  | *F*(2,370)=14.35^***^ | 16.9 | 38.9 | 22.8 | 13.4 | 8.0 |

*Note 1.* * p < .05; ** < .01; *** p < .001.

*Note 2*. 1 = *Not important*; 2 = *Unimportant*; 3 = *Important*; 4 = *Very important*; 5 = *Extremely important*.

*Note 3.* Criteria are presented only in self-report form. In the observer form, they were presented in third person with the subject ‘your son/daughter’.

**Table P.** Correlations between the same scales in self-report and observer form of youth emotional and behavioral problems (*N* = 125).

| **Youth Emotional and Behavioral Problems** | ***r*** |
| --- | --- |
| ***Broad-band scales*** |  |
| Internalizing | .45^*^ |
| Externalizing | .42^*^ |
| ***Syndrome scales*** |  |
| Anxious/Depressed | .42^*^ |
| Withdrawn/Depressed | .47^*^ |
| Somatic Complaints | .30^*^ |
| Delinquent Behavior | .24^*^ |
| Aggressive Behavior | .46^*^ |

*Note.* * FDR p < .05.

**Table Q.** Correlations between the same items of youth risk behaviors (YRB) in self-report and observer form (*N* = 124 if not otherwise indicated).

| **Youth Risk Behaviors (YRB)** | ***r*** |
| --- | --- |
| When you ride a bike, skateboard or overboard (etc.), how often do you wear a helmet?^a^ | .60^*^ |
| How often do you wear a seat belt when riding in a car? | .52^*^ |
| Have you ever ridden in a car driven by someone who had been drinking alcohol? | .17 |
| Have you ever carried a weapon, such as a gun, knife, or club? | - |
| Apart from a few sips, have you ever had a whole alcoholic drink? | .32^*^ |
| In the last 30 days, on the days you smoked, how many cigarettes (regular or electronic) per day have you smoked? | - |
| Have you ever engaged in self-harming behaviors (cutting yourself, scratching yourself, etc.) on a voluntary basis? | .17 |
| Have you ever been involved in a physical fight (e.g., you got into a fight, brawls, etc.)? | .33^*^ |
| Have you ever been bullied at school? | .59^*^ |
| Have you ever been electronically bullied (through texting, Instagram, Facebook, or other social media)? | .56^*^ |
| During the past 7 days, how many days have you eaten breakfast? | .62^*^ |
| When you have class, how many hours do you sleep per night? | .50^*^ |
| On a typical school day (even if in DAD), how many hours a day do you watch TV?^b^ | .63^*^ |
| On a typical school day (even if in DAD), on average, how many hours a day do you play video games or use computer or mobile phone for things not related to school? | .51^*^ |

*Note 1.* ^a^n = 80; ^b^n = 88.

*Note 2.* * FDR p < .05.

*Note 3.* Items are presented in self-report form. In the observer form, they were presented in third person with the subject ‘your son/daughter’.

*Note 4.* ‘-’ indicates that the correlations could not be calculated because at least one of the two variables was constant.

**Table R.** Correlations between the same items of passions, values, beliefs and behaviors (PVB&B) in self-report and observer form (*N* = 124).

| **Adolescents’ passions, values, beliefs, and behaviors (PVB&B)** | ***r*** |
| --- | --- |
| I believe in God. | .41^*^ |
| I believe that attending religious services (masses, weddings, funerals, etc.) is important. | .48^*^ |
| I prefer to believe what is said on social networks or on the Internet rather than what science says. | .36^*^ |
| I play a musical instrument, or I studied singing. | .64^*^ |
| I love animals. | .44^*^ |
| I prefer science to art. | .50^*^ |
| I listen to music. | .44^*^ |
| I write or I wrote a personal diary. | .52^*^ |
| I happened to write poems, stories, or books. | .42^*^ |
| I play sports. | .54^*^ |
| I was volunteering. | .23^*^ |
| I feel good. | .38^*^ |
| I make jokes or laugh at other people's jokes. | .43^*^ |
| I am or I was seriously engaged. | .26^*^ |
| When there is no school, I sleep late regardless of when I go to bed. | .58^*^ |
| I listen to older people with respect. | .44^*^ |
| I say 'please' and 'thank you'. | .32^*^ |
| I try to keep the promises I made. | .19^*^ |

*Note 1.* * FDR p < .05.

*Note 2.* Items are presented in self-report form. In the observer form, they were presented in third person with the subject ‘he/she’.

**Table S.** Correlations between the same items of the features of a possible future job (FFJ) in self-report and observer form (*N* = 124).

| **Questions regarding a possible future job (FFJ)** | ***r*** |
| --- | --- |
| It is important for you that the job is honest. | .16 |
| It is important for you that the job guarantees a good income. | .25^*^ |
| It is important for you that the job provides emotional security. | .18^*^ |
| It is important for you that the job allows you collaborate with other people. | .26^*^ |
| It is important for you that the job is organized and planned. | .34^*^ |
| It is important for you that the job allows you to be in contact with nature. | .33^*^ |
| It is important for you that the job allows you to be in contact with art. | .36^*^ |

*Note 1.* * FDR p < .05.

*Note 2.* Items are presented in self-report form. In the observer form, they were presented in third person with the subject ‘your son/daughter’.
